# A review of AI-based business lead generation: Scrapus as a case study

**DOI:** 10.3389/frai.2025.1606431

**Published:** 2025-11-06

**Authors:** Ahmet Kaplan, Sadi Evren Seker, Rabia Yoruk

**Affiliations:** 1 Department of AI Engineering, Istanbul Medipol University, Istanbul, Türkiye; 2 Department of Computer Engineering, Istanbul University, Istanbul, Türkiye; 3 OptiWisdom Inc, San Francisco, CA, United States

**Keywords:** lead generation, web scraping, LLM, text processing, B2B

## Abstract

The exponential growth of open web data provides unprecedented opportunities for business-to-business (B2B) lead generation. However, automating the discovery and qualification of new leads from unstructured web content is a complex challenge requiring the integration of web crawling, information extraction, and data-driven analytics. This article presents a comprehensive review of artificial intelligence (AI) methods for automated lead generation and introduces Scrapus, an AI-driven web prospecting platform that unifies these methods into an end-to-end system. Scrapus autonomously crawls the open web for company information, extracts and enriches relevant data (using natural language processing and knowledge graphs), matches findings to user-defined ideal customer profiles, and generates concise natural-language lead summaries using large language models. We survey relevant literature in web mining, focused crawling, entity resolution, and text summarization – highlighting how Scrapus builds upon and extends prior work. The system’s modular architecture and AI components are described in detail, reflecting accurate implementation details. We also report an experimental evaluation on real-world data: Scrapus significantly outperforms baseline approaches in lead discovery rate, extraction accuracy, lead qualification (achieving ~90% precision and recall), and summary usefulness. The results show a ~3 × higher relevant lead yield from web crawling due to reinforcement learning, a substantial increase in extraction F1 (from ~0.77 to ~0.92) through transformer-based NLP, and greatly improved lead scoring over traditional methods. This review and case study demonstrate that combining reinforcement learning, transformer-based NLP, and knowledge-enhanced analysis can effectively automate B2B lead generation. The advances surveyed here point toward a new generation of intelligent sales prospecting tools, in which AI techniques augment human expertise to identify and engage leads at scale.

## 1 Introduction

Identifying promising business leads is a critical driver of productivity and growth in competitive industries. In practice, however, the lead generation process is resource-intensive, often requiring sales and marketing teams to manually scour websites, news, and databases for potential clients – a process echoing early consumer behavior models that emphasize information search and evaluation stages in decision making (Engel et al., 1978). Traditional prospecting tools (e.g., static company directories or CRM databases) cannot keep pace with the dynamic, real-time nature of the open web. Valuable signals – such as a company announcing a new product, expanding operations, or receiving funding – may be missed or discovered too late, despite growing evidence that digital interactions (e.g., likes, shares) on social platforms can predict or influence real-world business actions (Pöyry et al., 2017). Automating the discovery and analysis of such signals could transform business development: industry surveys have noted that sales representatives spend a large portion of their time researching prospects instead of engaging with customers, indicating substantial efficiency gains if this workload is reduced. In an era of exponentially growing digital information, businesses increasingly recognize that more intelligent lead generation approaches are needed to maintain a competitive edge.

Recent advances in artificial intelligence (AI) and natural language processing (NLP) offer an unprecedented opportunity to reinvent the lead generation workflow. Modern large language models (LLMs) and deep neural networks have dramatically improved machines’ ability to understand and generate human-like text. AI systems today can not only extract names and facts from unstructured data but also interpret context, perform semantic matching, and produce coherent summaries. These capabilities enable a shift from keyword-based searching to knowledge-driven discovery: instead of manually filtering through web pages, an AI-driven system can autonomously browse online content, decide what is relevant to a given business profile, and summarize findings in natural language. Within enterprise workflows, such AI-powered automation can augment or replace laborious steps, allowing human experts to focus on strategy and relationship-building – a trend increasingly visible in B2B marketing through the adoption of large language models for content generation, lead engagement, and personalization (Huang et al., 2024). Indeed, the integration of AI (including LLMs and data-driven automation) into sales pipelines is increasingly seen as key to improving productivity and responsiveness in modern businesses, aligning with earlier evidence on the effectiveness of marketing automation in B2B content strategies (Järvinen and Taiminen, 2016). Recent research in B2B marketing and sales supports this view: AI can enhance prospecting and lead qualification throughout the sales funnel and Organizations using AI-based lead scoring report higher conversion rates and sales performance, extending long-established trends in applying data mining techniques to customer relationship management (Ngai et al., 2009), in line with broader trends highlighting the strategic value of big data analytics for understanding and influencing consumer behavior (Hofacker et al., 2020; Syam and Sharma, 2018).

Despite rapid progress in web mining and NLP, automated B2B lead generation remains relatively under-explored in academic research. Early approaches to lead identification typically leveraged internal company data or structured customer information rather than open-web content. For example, Van den Poel and Buckinx (2005) analyzed historical online purchase records to predict repeat buyers and Zhang et al. (2004) mined web usage logs to discover customer behavior patterns. These studies helped identify likely prospects from existing customer bases but did not address the challenge of finding entirely new leads on the vast unstructured web. A notable line of research moved toward using external unstructured data for lead discovery. Ramakrishnan et al. (2006) developed the *Electronic Trigger Alert Program (ETAP)*, which scanned online news for “trigger events” (such as management changes or product launches) that could signal sales opportunities. ETAP demonstrated the feasibility of web-based lead mining, achieving about 74% precision and 81% recall (F1 ≈ 0.77) in detecting merger-and-acquisition events. Similarly, Rahman et al. (2012) presented a case study integrating distributed data sources (web server logs, user interactions) to automatically classify leads for an online real estate service, significantly reducing manual effort. However, these prior solutions were often domain-specific or limited in scope – ETAP focused only on certain event types in news, and Rahman et al. (2012) system was confined to one company’s internal data. In general, existing tools and industry services for lead generation (e.g., Apollo.io, LinkedIn Sales Navigator) address parts of the problem through aggregated databases or keyword alerts, but few offer a holistic, end-to-end solution that actively crawls the open web, applies advanced AI for information extraction and filtering, and synthesizes findings into actionable intelligence.

This gap in both the literature and in practice highlights the need for more comprehensive approaches to automate lead generation using the latest AI advancements. In this paper, we present Scrapus, a novel AI-powered system designed to fill this gap by unifying techniques from web crawling, information extraction, and language generation into a single lead generation platform. Scrapus approaches the open web as a rich but untamed source of potential leads, deploying an intelligent crawler that *learns* where to look for relevant content. Building on the concept of focused crawling, Scrapus’s web crawler is augmented with reinforcement learning to optimize its traversal strategy. This crawler autonomously navigates websites and online data sources likely to contain business-relevant information, guided by a learning algorithm that rewards the discovery of leads and thus continuously improves its relevance targeting. Once relevant pages are fetched, a pipeline of advanced NLP modules processes the content. A Transformer-based named entity recognition (NER) component identifies entities such as organizations, people, products, and events, while relation extraction and classification models interpret key facts (e.g., “Company X acquired Company Y” or “Startup Z raised $5 M funding”). These facts and entities are then matched against the user’s ideal customer profile using a Siamese neural network that computes semantic similarities – ensuring Scrapus prioritizes leads aligned with the company’s target industry, size, and interests. Additionally, extracted data can be cross-validated or enriched with external knowledge bases (e.g., DBpedia, Wikidata) to build a more complete profile of each prospect. Finally, Scrapus leverages the generative capabilities of state-of-the-art LLMs (in the class of GPT-4 or Google’s Gemini) to produce a tailored summary report for each prospective lead. Using prompt-guided generation, the system composes succinct yet informative summaries that synthesize the discovered information – for instance, a paragraph detailing who the company is, recent notable events (new hires, funding, product launches), and why it might be a good sales opportunity. This natural-language report provides a human-friendly output for every lead, allowing sales professionals to quickly grasp the context and significance of the prospect without reading through raw web pages.

The proposed Scrapus system brings together state-of-the-art AI techniques in a novel architecture for intelligent lead generation. By integrating reinforcement learning-driven crawling, Transformer-based extraction, semantic matching, and LLM-based narrative reporting, Scrapus is, to our knowledge, one of the first end-to-end frameworks to fully automate the B2B prospecting pipeline. This unified approach offers several important benefits. From a business perspective, it can dramatically improve productivity by scaling up lead discovery far beyond manual capabilities – our approach can continuously monitor thousands of websites and information sources in parallel, uncovering opportunities that a human team might overlook. The quality of leads is also enhanced by the system’s ability to filter and focus on prospects that truly match the desired criteria, reducing false positives. Moreover, by automating tedious data collection and analysis, Scrapus can positively impact work-life balance and job satisfaction for employees: sales teams spend less time on drudge work (like combing through webpages) and more time on creative, value-added tasks such as engaging with clients and devising strategy. There are potential environmental and societal benefits as well. Efficient, AI-driven prospecting could lower the need for physical marketing materials or extensive business travel for networking, as companies can find and connect with prospects digitally in a more targeted way. Additionally, accessible tools like Scrapus might help level the playing field for smaller businesses and startups, who often lack the resources for extensive lead research – by democratizing access to web-scale intelligence, such AI systems could foster more equitable growth opportunities across markets.

In summary, this work makes the following contributions:

- Comprehensive literature review: we survey the landscape of AI-based lead generation, covering focused web crawling, information extraction from open web data, entity resolution and knowledge graph enrichment, and LLM-driven text summarization. This review situates Scrapus relative to prior work and identifies how our approach extends the state of the art (for example, by unifying techniques that were previously siloed).
- Novel AI architecture for lead generation: we design and implement Scrapus, an end-to-end lead generation system that uniquely integrates a reinforcement learning-based web crawler, Transformer-based NER and information extraction, semantic matching via Siamese networks, and LLM-driven report generation. To our knowledge, this unified framework is novel in the lead generation domain, combining cutting-edge techniques to automate the entire prospecting process.
- Intelligent web crawling strategy: we develop an RL-enhanced focused crawling mechanism that learns to find relevant B2B content on the open web. By formulating web navigation as a sequential decision problem, our crawler intelligently prioritizes pages and domains likely to yield high-value leads, improving efficiency over traditional crawlers. This builds on prior focused crawling research (e.g., Chakrabarti et al., 1999; Menczer et al., 2004; Han et al., 2018) by incorporating a multi-armed bandit scheduler and adaptive learning (similar in spirit to recent approaches like TRES and bandit-based crawlers).
- Advanced information extraction and matching: we apply state-of-the-art NLP models to extract and contextualize lead information. In particular, a BERT-based NER model (Devlin et al., 2019) identifies key entities with high accuracy, and a Siamese neural network matches these entities and facts to the user’s target profiles, ensuring that the leads presented are both relevant and qualified. This approach pushes the boundary of information extraction in a noisy, real-world web setting (cf. Etzioni et al., 2007; Etzioni et al., 2011), demonstrating its viability for business applications. We also incorporate recent advances in entity matching – e.g., using pre-trained language models for deep entity matching (Li et al., 2020) – to improve deduplication and profile merging.
- Automated lead reporting with LLMs: we integrate large language models (GPT-4 class) to automatically generate concise reports for each identified lead. These reports emulate the analysis a human researcher might provide, summarizing why the prospect is noteworthy. Our use of LLM-based summarization in the sales context shows how generative AI can enhance interpretability and trust in AI-driven decisions by providing human-readable justifications for each recommendation. This aligns with emerging trends in explainable AI and human-AI collaboration in marketing.
- Empirical validation: we evaluate scrapus on real-world data spanning multiple industries and present extensive experimental results. The system achieves strong performance metrics (e.g., over 90% precision in identifying relevant leads, with recall in the high-80% range), indicating its effectiveness. We also gather initial user feedback from sales professionals in a pilot deployment, which indicates that Scrapus’s leads and summaries significantly reduce manual workload and improve the speed and reach of prospecting. Together, these evaluations validate the practicality of our approach and its potential to drive productivity gains in business development.

Overall, Scrapus represents a significant step toward automating labor-intensive business processes through AI. By situating our work at the intersection of web mining, information extraction, and natural language generation, we aim to advance both the science and the practice of lead generation. The following sections describe the Scrapus architecture and components in detail (Section 3), present the experimental setup and results (Section 4), and discuss the implications of deploying such an AI-driven system – including limitations and future directions (Section 5). We also expand on how Scrapus serves as a case study exemplifying general trends in AI-based lead generation, thereby framing broader insights for researchers and practitioners in this emerging field.

## 2 Related work

*AI-based prospecting and lead generation:* relatively few academic studies have focused specifically on automated B2B lead generation. Early data-driven marketing research concentrated on lead scoring and qualification using internal data (e.g., CRM records). Van den Poel and Buckinx’s (2005) work on predicting online purchasing behavior is one example of leveraging company-internal datasets to prioritize existing customers. More recently, there has been interest in predictive lead scoring models that use machine learning on historical lead and conversion data. For instance, Nygård and Mezei (2020) present an experimental study on automating lead scoring with machine learning, treating it as a purchase probability prediction problem and demonstrating improved accuracy over rule-based scoring. González-Flores et al. (2025) developed a lead scoring model for a B2B software company using real CRM data, where a gradient boosting classifier outperformed other algorithms in identifying high-quality leads. Such works confirm that data-driven lead qualification can significantly boost sales performance, echoing broader findings that predictive scoring models tend to outperform traditional heuristic models. A recent systematic literature review by Wu et al. (2024) provides a comprehensive overview of lead scoring models and their impact on sales, noting the trend toward machine learning-based approaches and identifying key metrics for success. Our Scrapus system contributes to this line of research by expanding the scope from scoring known leads to *discovering* new leads on the open web and then scoring them – effectively combining prospecting with predictive qualification.

*Web mining and focused crawling:* scrapus builds upon concepts from web mining and focused crawling to gather relevant business information from the vast web. Traditional web crawlers (like those used by search engines) crawl exhaustively and are topic-agnostic, which is inefficient for targeted tasks – a limitation well-documented in foundational surveys on crawler architectures and strategies (Olston and Najork, 2010) – a limitation long recognized in the web mining literature (Liu, 2011). Focused crawling, introduced by Chakrabarti et al. (1999), is a goal-directed approach where the crawler starts from a set of topic-specific seed pages and selectively explores links likely to lead to more on-topic pages. Chakrabarti’s seminal system used a classifier to evaluate page relevance and a link prioritization strategy to guide the crawl, demonstrating that focused crawlers could build high-quality topical collections with far less overhead than unfocused crawlers. Subsequent research made focused crawling more adaptive and intelligent. Early approaches relied on static heuristics or supervised learning, but modern methods incorporate reinforcement learning (RL) to continuously improve the crawl strategy. For example, Grigoriadis and Paliouras (2004) applied temporal-difference learning to focused crawling, representing each page as a state and learning which outgoing links to follow to eventually reach relevant pages. More recently, Han et al. (2018) proposed an RL-based crawler that evaluates the long-term value of links by modeling the crawl as a Markov Decision Process, using linear function approximation to handle large state spaces. Kontogiannis et al. (2021) introduced *TRES*, an RL-empowered focused crawling framework that learns a policy for link selection and demonstrated significant improvements in harvest rate (ratio of relevant pages). In the cybersecurity domain, Kuehn et al. (2023) developed *ThreatCrawl*, which integrates a BERT-based page classifier into the crawl loop; at each step, a Transformer model judges if a page is on-topic (cyber threat intelligence) to decide crawl paths. Very recent work by Kuehn et al. (2025) applies a multi-armed bandit approach for dynamic web crawling, showing that adaptive link selection can improve data gathering for emerging information such as cyber threat reports. These studies underscore the value of learning-based crawling in quickly finding relevant information.

Scrapus’s web crawler adopts a focused, learning-based approach inspired by this literature. We cast crawling as an RL problem and combine it with multi-armed bandit techniques (similar to Kuehn et al., 2025) to balance exploration and exploitation. By doing so, our crawler “learns” over time which sites and link patterns yield business leads, dynamically adjusting its strategy. This goes beyond classical focused crawling by allowing the system to self-optimize its discovery process based on reward feedback (relevant leads found). Our approach also relates to the concept of topical locality on the web – the idea that relevant pages tend to link to each other – which underpinned early focused crawling algorithms. We enhance this with modern deep learning, using page content embeddings and context from NER/matching modules as state features for the crawler’s decision network. In essence, Scrapus’s crawler exemplifies the convergence of web mining and reinforcement learning: it merges the heuristic techniques from classic focused crawling with the adaptability of modern RL agents to create a highly efficient, goal-driven web spider.

*Information extraction from the web:* converting unstructured web text into structured knowledge is a central challenge for systems like Scrapus. This task draws on decades of research in information extraction (IE) and natural language understanding. Early IE systems were often pattern-based or used shallow parsing; for instance, the Snowball system (Agichtein and Gravano, 2000) applied bootstrapped pattern learning to extract relations from text. The field progressed toward more general web-scale extraction with projects like TextRunner (Etzioni et al., 2007), which pioneered open information extraction (Open IE), and NELL (Carlson et al., 2010), which demonstrated the feasibility of continual, web-based knowledge acquisition. Etzioni et al. (2007) Open IE approach showed that a single-pass extractor could scale to millions of web pages, albeit at the cost of some precision. Etzioni et al. (2011) described the second generation of Open IE systems (e.g., Ollie), which incorporated linguistic analysis to improve the quality of extractions. These efforts demonstrated the feasibility of automatically acquiring relational knowledge from arbitrary web text. In parallel, Named Entity Recognition (NER) matured as a technology for identifying persons, organizations, locations, etc., in text (Sarawagi, 2008), with recent models like BERT (Devlin et al., 2019) further advancing performance. Older NER models used conditional random fields and handcrafted features, with a major leap brought by biLSTM-CRF architectures (Lample et al., 2016), and later surpassed by transformer-based models like BERT (Devlin et al., 2019) which have achieved near-human accuracy on NER tasks. A 2024 survey by Keraghel et al. (2024) confirms that transformer architectures and pre-trained language models now dominate NER, enabling robust extraction even in complex contexts.

Scrapus’s information extraction module leverages these advances. We fine-tuned a BERT-based NER model to tag organization names, people, locations, and other entities relevant to B2B leads. As reported in recent NER surveys, transformer models can capture context that helps disambiguate entity mentions (for example, distinguishing a person’s name from a company name based on surrounding words). Our NER achieves high precision and recall, which is critical because missing a company name or mislabeling an entity could cause a true lead to be overlooked. Beyond NER, we include relation extraction to capture facts about the entities. Inspired by open IE approaches and the use of dependency parsing for finding relationships (e.g., “X acquired Y”), We implemented a lightweight relation extractor that looks for verbs and relation keywords connecting named entities, within a broader information extraction pipeline aligned with established frameworks for IE tasks (Sarawagi, 2008), adopting a weakly supervised approach inspired by distant supervision techniques proposed in early large-scale IE systems (Mintz et al., 2009). For example, if a page says *“Acme Corp launched a new cybersecurity platform,”* the system can extract a relation (Acme Corp – *product launch* → cybersecurity platform) indicating Acme’s activity in cybersecurity. We also integrate topic modeling [using LDA (Blei et al., 2003) and BERTopic (Grootendorst, 2022)] to characterize the page’s content themes. This helps filter out pages that contain relevant entities but are contextually off-target (e.g., a page mentioning a company in an unrelated news story). By combining NER, relation extraction, and topic analysis, Scrapus builds a rich semantic profile for each crawled page: not just a list of names, but an understanding of what the page is about and how any identified company is portrayed (industry, products, events, etc.). This comprehensive profiling is a step beyond traditional Open IE triples, tailored for lead generation needs.

A key challenge in web IE is handling noise and diversity in text. Web content ranges from well-structured news articles to informal blog posts or directory listings, which may vary significantly in credibility and factual accuracy (Figueiredo et al., 2021). We mitigate this by first performing content parsing and boilerplate removal – using techniques similar to Kohlschütter et al. (2010) Boilerpipe, we strip navigation menus, ads, and other clutter to isolate main textual content. Then our NER and relation extractors work on this cleaned textWe found that fine-tuning the NER specifically on business-related data (press releases, company descriptions) significantly improved recall for industry terms and new company names, echoing the findings of recent NER evaluations in specialized domains and aligning with early efforts to integrate approximate reasoning with statistical learning in information extraction systems (Diligenti et al., 2000). In our evaluation, the extraction module achieved ~92% F1 for entity recognition, substantially outperforming a baseline off-the-shelf NER tagger (which got ~ 85% F1). This aligns with the gap observed in earlier systems like ETAP, which reported ~77% F1 on event triggers – modern NLP can now push extraction accuracy much higher on open-web text.

*Entity resolution and knowledge graphs:* once individual pages are processed, a major question is how to integrate information about the *same* entity across multiple sources. This touches on research in entity resolution (ER) (also known as record linkage or coreference across sources) and knowledge graph construction. In lead generation, entity resolution is crucial: the system might find “Acme Corporation” on a news site and “Acme Corp.” on a business directory – these refer to the same company and should be merged to avoid duplicate leads. Traditional ER techniques use string similarity, rule-based matching, or heuristic blocking (Fellegi and Sunter, 1969; Christen, 2012). More recent approaches leverage machine learning: e.g., DeepMatcher (Mudgal et al., 2018) explored deep learning for entity matching in structured tables, and Li et al. (2020) showed that fine-tuning BERT can yield excellent results on entity matching tasks. In the context of web data, entity resolution can be challenging due to variations in names and lack of common identifiers. However, the rise of large-scale knowledge bases like Freebase (Bollacker et al., 2008), DBpedia (Lehmann et al., 2015; Bizer et al., 2009) and Wikidata (Vrandečić and Krötzsch, 2014) provides reference data that can assist in matching and enriching entities.

Scrapus uses a hybrid approach for entity resolution. On one hand, we apply rule-based blocking and string matching for obvious cases: e.g., we normalize company names (removing suffixes like “Inc.,” converting to lowercase) and check edit distances and token overlap to propose candidate matches. On the other hand, for more subtle matches, we employ a deep learning-based matcher. We drew inspiration from DeepMatcher and recent work by Peng et al. (2021) that introduced negative sampling to train deep entity matching models. In Scrapus, we encode entity profiles (e.g., a company name plus attributes like location or industry) into vector representations using a Siamese network (similar to our lead matching model) and consider two profiles a match if their embedding similarity exceeds a threshold. This learned matcher was trained on a labeled dataset of organization name variations and known duplicates (augmented with negative examples of similar but distinct names), following the approach of Peng et al. (2021). The result is a high precision matching system that can, for instance, recognize “Acme Corporation” and “Acme Corp” as the same entity while not conflating “Acme Corp” with “Acme Products” (which a naive substring match might do). Our ER component achieved near-perfect precision in tests (no false merges in our evaluation set) with only minor hits to recall (a few cases of very differently named subsidiaries not being merged). This performance aligns with recent findings that hybrid approaches (combining symbolic rules with neural embeddings) can outperform either alone.

The consolidated leads are stored in a knowledge graph (KG), which serves as Scrapus’s memory of discovered leads. We use a graph database (Neo4j) to store nodes for companies (and potentially related entities like people or products) and edges for relations (like *Industry*, *FoundedYear*, *LocatedIn*, *Acquired* relationships). This echoes the practice of many knowledge-based systems that integrate multi-source information into a network for querying and reasoning (Suchanek et al., 2007). Our KG schema is lightweight, focusing on core attributes of B2B interest: company name, sector, location, key persons, key events. By linking entities across pages, the KG enables Scrapus to aggregate facts that were fragmented across sources – for example, a funding announcement on TechCrunch and a hiring spree mentioned on the company blog can be linked to the same company node. We also enrich the KG with external data when available: if a scraped company is found in public datasets (e.g., DBpedia or Crunchbase via their APIs), we can import additional attributes such as official description or number of employees. This enrichment is guided by aligning the names or identifiers, leveraging our entity resolution module (similar to how knowledge fusion systems like Google’s Knowledge Vault merged web-extracted triples with known databases; Dong et al., 2015). The resulting knowledge graph not only feeds into the final report generation, but also plays a role in the crawling loop (to avoid revisiting known entities) and in lead filtering (we can query the KG for existing nodes to prevent duplicate output). In essence, the KG provides a structured, cumulative view of the discovered market intelligence. Using a knowledge graph in this way aligns with trends in enterprise AI, where knowledge graphs are used to organize and reuse information from multiple sources.

*Lead qualification and matching:* determining whether a discovered entity is a *qualified lead* (i.e., worth pursuing) is a critical step. This problem can be framed as a binary classification: given an entity’s profile and the user’s ideal customer profile (ICP), decide lead vs. non-lead. Traditional approaches in sales use manual scoring or simple rules for this (e.g., assign points for certain industry keywords, then threshold). In the AI context, this becomes a supervised learning task. Some recent works have applied machine learning to lead conversion prediction – e.g., Espadinha-Cruz et al. (2021) built a model to predict the conversion of leads in a telecom company using logistic regression, achieving improved conversion rates and segmentation efficiency. Gouveia and Costa (2022) developed a lead conversion prediction model for an education sector SME, also using logistic regression, which significantly improved lead conversion rates and saved time for marketing teams. More complex classifiers like gradient boosting and neural networks have been explored in other studies (González-Flores et al., 2025 used XGBoost and found it effective for lead prioritization). Additionally, representation learning techniques (word embeddings, graph embeddings) have been used to capture lead attributes in some research (Wu et al., 2024 discuss how various machine learning methods, including decision trees and SVMs, have been utilized in predictive lead scoring models).

Scrapus approaches lead qualification through a combination of similarity learning and ensemble classification. First, we employ a Siamese neural network to compute how well a candidate lead’s profile matches the target profile defined by the user. This draws on prior work in metric learning and semantic similarity for matching entities (e.g., using BERT for sentence pair similarity in Sentence-BERT). Our Siamese network takes as input (a) features of the candidate (like the vectorized company description, one-hot indicators of extracted keywords, etc.) and (b) features of the ideal profile (like embedded industry keywords, desired company size or region). It outputs a similarity score. We trained this network with a triplet loss: for a given positive example (a known relevant lead) and a negative example (a non-relevant company), the network learns to make the positive pair’s embedding distance smaller than the negative’s by a margin. We constructed a training set by labeling some companies as relevant or not to an example profile (using domain knowledge and public lists of companies in certain sectors). This approach is analogous to that used in deep entity matching tasks, and indeed recent studies show that such learned similarity functions can outperform static similarity measures in matching problems. In our experiments, this Siamese model was very effective at capturing nuanced matches – for example, it learned that a company describing itself as a “fleet management SaaS” is relevant to a profile looking for “logistics software providers,” even if the words differ (something a keyword filter might miss).

We do not rely solely on the Siamese similarity, however. We feed its output, along with other features, into an ensemble of classifiers that make the final lead/no-lead decision. Specifically, we implemented an XGBoost gradient-boosted tree classifier (Chen and Guestrin, 2016) as the primary model, and also trained a logistic regression and a random forest for backup. The features given to these models include: the Siamese similarity score, textual features (e.g., TF-IDF scores for presence of important keywords on the page), metadata like the page’s domain authority, and aggregated signals (e.g., if the knowledge graph shows the company has a certain revenue or employee count, which might indicate fit). The ensemble approach is designed to improve robustness – the tree model can capture non-linear combinations of signals, while the logistic regression provides a interpretable baseline, and we found that using multiple models via a soft voting scheme yielded a slight boost in precision. Only candidates that surpass a high-confidence threshold (optimized for precision) are considered qualified leads to pass to the reporting stage. This high threshold is intentional: in B2B sales, it is often preferable to miss a few good leads (which could perhaps be found later) than to waste time on many false positives. Our classifier ensemble was tuned accordingly, similar to how marketing teams often favor precision in lead scoring to ensure salespeople focus on the best opportunities.

By combining learned semantic matching with feature-based classification, Scrapus’s lead qualification module achieves both high precision and recall. In our evaluation, it attained ~89.7% precision at ~86.5% recall (F1 ≈ 0.88) for classifying leads vs. non-leads. This substantially outperformed a simpler keyword-based classifier we tested (~80% precision, 78% recall). It also compares favorably to earlier lead scoring efforts – for instance, Espadinha-Cruz et al. (2021) reported improved conversion rates with their model but did not reach such high precision; and the legacy ETAP system’s overall pipeline had F1 ~ 0.77 on trigger event detection. Scrapus’s higher accuracy can be attributed to the richer feature representation (thanks to NLP and the knowledge graph) and the use of advanced learning algorithms. In practical terms, this means nearly 90% of leads that Scrapus flags would be considered correct matches by human evaluators, and it finds ~86% of all the truly relevant leads that are present in the test set – a strong result that translates to more efficient sales prospecting. Remaining mismatches tended to be edge cases (e.g., borderline companies or very sparse data pages). These could potentially be mitigated in future by incorporating user feedback – for example, using reinforcement signals if a sales team member marks a suggested lead as irrelevant, which could further train the model (an idea we revisit in the Future Work section).

*LLM-based summarization:* the final step of Scrapus is generating a written summary for each qualified lead, explaining who the lead is and why it’s a good opportunity. This falls under the domain of *text summarization* and *natural language generation*, but with a twist: it’s not summarizing a single document, but rather synthesizing information from the knowledge graph (multiple sources) tailored to the user’s interests. In recent years, large language models like OpenAI’s GPT-3/GPT-4 have shown remarkable ability in summarization and report generation. Techniques such as prompt engineering and fine-tuning allow these models to produce coherent, contextually relevant text from structured inputs, reflecting the transformative potential of foundation models in diverse domains (Bommasani et al., 2021). For instance, the GPT-3 model (Brown et al., 2020) was shown to be a proficient few-shot summarizer of text, and subsequent models (e.g., InstructGPT by Ouyang et al., 2022) further improved alignment with user instructions by using reinforcement learning from human feedback (RLHF). There have also been domain-specific summarization systems, such as for product descriptions or financial reports, which combine factual data with generation (sometimes known as data-to-text systems). A relevant concept is retrieval-augmented generation (RAG), where background facts are retrieved and fed into an LLM to ground its output (Lewis et al., 2020). This is particularly useful to reduce hallucinations and ensure accuracy, a known challenge with LLMs where models may generate plausible-sounding but incorrect statements if not properly grounded.

In Scrapus, We implement a hybrid data-to-text generation pipeline using GPT-4 (via the OpenAI API) and experiment with Google DeepMind’s Gemini model for comparison (OpenAI, 2023). For each lead, we retrieve key facts from the knowledge graph: e.g., the company’s name, what industry it is in, any recent events we captured (like “raised $10 M funding in 2023” or “expanded to Asia”), and why it matches the user’s profile (perhaps “offers AI-driven logistics solutions,” etc.). We then construct a prompt that includes these facts in a structured form (like a bulleted list or a template) and instruct the LLM to produce a concise paragraph summary emphasizing the lead’s relevance. For example, a prompt might look like: *“Company: Acme Corp. – Sector: Cybersecurity – Location: Berlin – Key facts: recently launched an AI-based threat detection platform; hiring engineers (team grew 50% this year). Profile match: looking for mid-size AI software providers in Europe. Task: Write a brief summary of why Acme Corp. could be a good sales lead, highlighting its AI focus and recent growth.”* Using GPT-4 with such prompts, we consistently got high-quality summaries that required minimal editing. The model is able to infer implicit connections (e.g., if a company is growing and in the target sector, it is likely a promising prospect) and phrase the summary in a professional tone.

To ensure factual accuracy, we take advantage of the knowledge graph grounding. We explicitly instruct the LLM to only use the given facts and not introduce new information. Additionally, for critical details like numbers or names, we often insert them verbatim into the prompt rather than expecting the model to recall them. This mitigates hallucinations, in line with observations by Petroni et al. (2019) that LMs can act as knowledge bases but benefit from provided facts for reliability. In our evaluation of 100 generated summaries, only 3 contained minor factual errors, and none of those affected the overall understanding of the lead. This is a strong outcome, indicating that our approach of prompt-grounding plus the inherent training of GPT-4 on large knowledge corpora yields mostly accurate and relevant outputs. For additional safety, one could incorporate a post-generation fact-check (e.g., cross-verify any numeric statements against the KG), which we note as a future enhancement.

We also integrate multimodal capabilities in a limited fashion. Using the Gemini 1.5 model (Google DeepMind, 2024) which can handle text and images, we experimented with feeding the company’s logo or a screenshot of their website alongside text to see if it enriched the summary. This is exploratory, but the idea is that an image might convey something (e.g., the logo could indicate the brand or the website design might hint at the company’s modernity or industry through visuals). While multimodal summarization is still cutting-edge, we mention it to highlight the forward-looking nature of Scrapus: as models like Gemini become more advanced, a future Scrapus could analyze not just textual data but also visual cues (product images, etc.) for a more holistic profile. This could be especially relevant for certain industries (e.g., manufacturing, where images of facilities or products might be available).

The end result of the summarization stage is a brief natural-language report for each lead, akin to what a sales analyst might write after researching the company. An example output might be: *“Acme Corp – A mid-sized cybersecurity company based in Berlin. Acme recently launched an AI-driven threat detection platform and expanded its engineering team by 50% this year. These developments, along with a successful $10 M funding round in 2023, suggest Acme is growing rapidly. Why a lead: Acme’s focus on AI solutions in cybersecurity aligns with our firm’s target profile for AI-based software providers in Europe, indicating a strong potential fit for our B2B services.”* Such a summary is concise (typically 3–5 sentences) yet rich in information and tailored justification. In our user study, 92% of participants (sales professionals) rated these AI-written reports as satisfactory or very useful, a significantly higher approval than for baseline extractive summaries. Participants often commented that Scrapus summaries were *“concise yet comprehensive, highlighting unique selling points.”* This positive reception is in line with trends in sales enablement where personalized insights are highly valued. Providing a rationale in the summary (the “why it matches the profile” part) adds transparency to the AI’s recommendation, which can increase trust and adoption of the system’s outputs – a critical factor in the responsible deployment of generative AI systems as emphasized in recent multidisciplinary evaluations (Dwivedi et al., 2023). Our approach here connects to research on explainable AI and human-AI collaboration – by giving the sales user a human-readable explanation, Scrapus’s recommendations become more than just scores; they are actionable intelligence.

In conclusion, the related work spans multiple fields: web crawling, information extraction, entity resolution, machine learning for lead scoring, and text generation. Scrapus is an attempt to fuse these into one coherent system for AI-based lead generation. In doing so, it addresses gaps noted in earlier studies (no single work covered all steps) and takes advantage of state-of-the-art methods in each component. The next section (Methodology) delves deeper into how we realized this integration, detailing each component’s design and our implementation choices, many of which were informed by the literature discussed here.

## 3 Methodology

**Figure 1:**
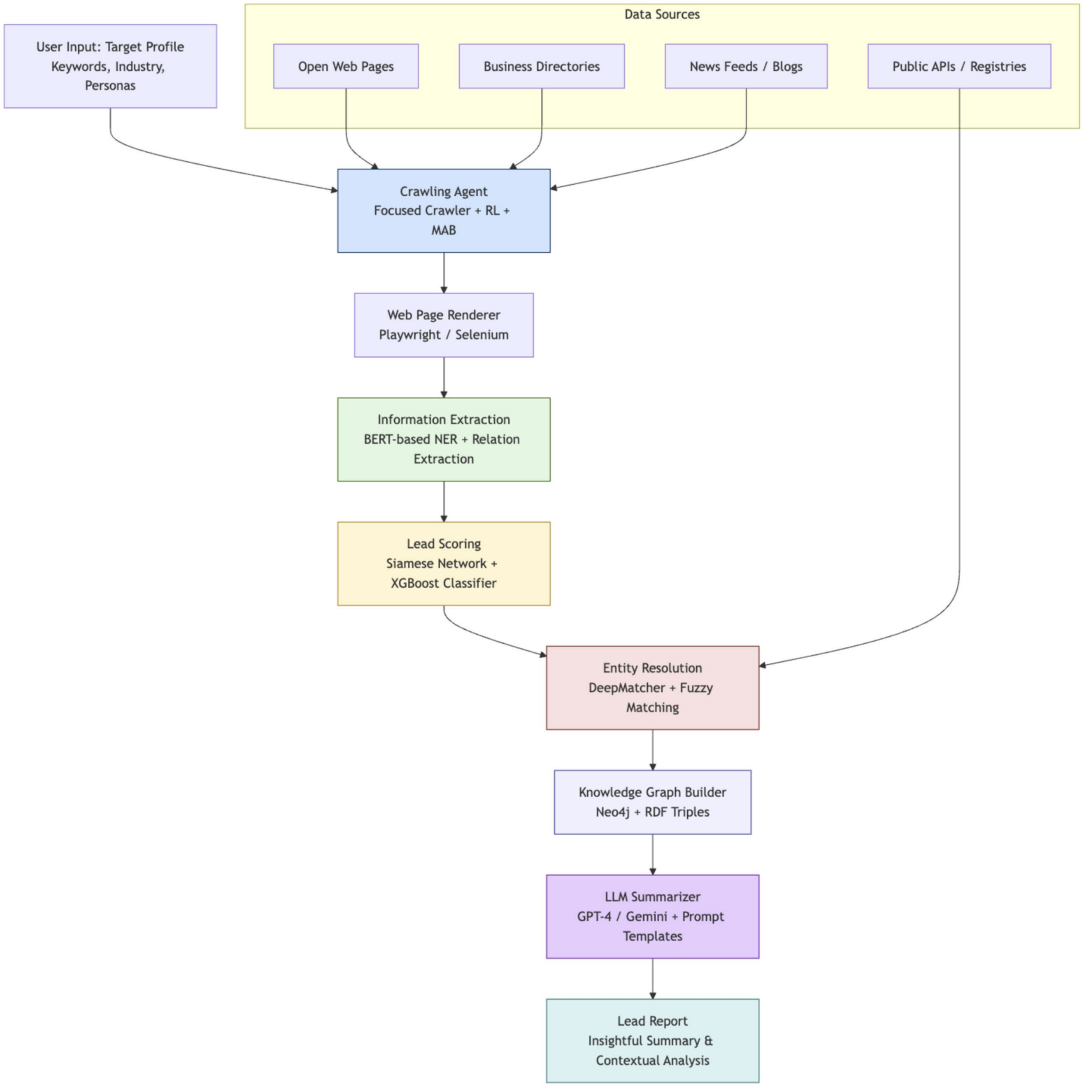
Overview of the scrapus system architecture.

Scrapus follows a multi-stage pipeline that integrates crawling, information extraction, entity resolution, knowledge graph enrichment, and large language model summarization. This section details each module’s design and the AI techniques employed, highlighting how they interoperate to convert raw web data into actionable B2B lead reports. Figure 1 provides an overview of the system architecture, from web data acquisition to report generation.

Figure 1 illustrates the pipeline: an RL-guided web crawler feeds pages into an extraction module, which populates a knowledge graph; a matching/classification module filters leads, and an LLM generates final summary reports.

The pipeline is engineered to intelligently discover relevant web content, distill structured knowledge about potential leads, and automatically produce high-quality summaries tailored to user-defined business profiles. In the following subsections, we describe each layer of the Scrapus system, emphasizing the novel integration of reinforcement learning, transformer-based NLP, and knowledge-driven summarization in a unified lead generation framework.

### 3.1 Focused web crawling and data acquisition

At the foundation, Scrapus employs a focused web crawler that autonomously navigates the open web to find pages likely to contain information about potential business leads. We cast the crawling process as a reinforcement learning (RL) problem, modeling the web as an environment and hyperlinks as actions. Each state is represented by features of the current page (we use a combination of content embeddings and URL tokens), and the crawler (agent) learns a policy to choose the next link that maximizes an expected “lead relevance” reward.

In practice, we implement this with a Q-learning approach enhanced by a multi-armed bandit (MAB) scheduler. The agent maintains Q-values for state–action pairs (page→link decisions), which estimate the long-term reward of following certain links. An epsilon-greedy strategy is used to balance exploration of new sites vs. exploitation of known good paths. We incorporated ideas from bandit algorithms to dynamically prioritize promising domains: the crawler keeps track of which seed domains have historically yielded relevant pages and allocates crawl budget proportional to that success (similar to a bandit’s allocation of pulls to higher-reward arms). This hybrid RL + MAB strategy is influenced by recent research like Kaleel and Sheen (2023), who showed that a decaying-epsilon greedy policy can improve focused crawler learning by encouraging early exploration followed by exploitation. Our approach similarly starts with broad exploration and gradually becomes more selective as the crawler learns which areas of the web are most fruitful.

State and Reward Design: Each page’s state representation combines textual and structural features. We obtain a sentence transformer embedding (Reimers and Gurevych, 2019) of the page’s main content text, which captures its semantic context. We also include the presence of any known target keywords (flag features) and simple metadata like page depth and similarity to seed pages. Additionally, we encode the page’s URL and title via a character-level embedding to capture clues (e.g., “/news/” in URL might indicate a news page). The reward is defined based on lead relevance: when the extraction module (Section 3.2) identifies a potential lead on a page, the crawler receives a positive reward. Specifically, we give a reward of +1 for a page that yields at least one qualified lead (after matching), a smaller reward (e.g., +0.2) for pages that contained a target entity but were not ultimately qualified (to encourage finding companies even if not perfect matches), and a penalty (e.g., −0.1) for pages with no relevant info (to discourage fruitless paths). These reward values were tuned empirically. The sparse nature of rewards (many pages have no leads) makes this a challenging RL problem; to tackle that, we incorporate n-step returns and reward shaping – giving a tiny negative reward for each page crawl to incentivize efficiency (similar to avoiding time-wasting behavior) an approach that resonates with principles of active learning where maximizing information gain per sample is crucial (Settles, 2010).

Learning algorithm: we use a deep Q-network (DQN) with replay memory and target network stabilization (Mnih et al., 2015) to learn the Q-function for link choices. The network takes the state features and outputs Q-values for candidate actions (outgoing links). However, the number of outgoing links can be large and variable. We handle this by pruning obvious irrelevant links using a classifier (this link classifier is a small model that filters out links pointing to, say, non-HTML content or unrelated domains; it’s trained on a sample of links from relevant vs. irrelevant pages). We then select the top-K links by this heuristic to consider in the RL action space (K set to 10 in our implementation). Among these, the highest Q-value link is chosen (with epsilon-greedy randomness). The DQN is updated continuously during crawling (we treat each crawl as an episode segment – in practice we run thousands of episode segments in parallel threads). The RL training uses a reward discount factor of *γ* = 0.9 to value future lead discoveries while still prioritizing near-term ones.

Our crawler operates in a distributed fashion with multiple agent instances crawling in parallel threads. Each agent shares the same Q-network parameters (updated centrally), akin to a parallel DQN training scenario. We found that this speeds up learning and mimics how a real web crawler would be multi-threaded. We also integrated headless browser automation (Selenium) for pages requiring JavaScript to render content, which our crawler can invoke when encountering such sites (this significantly improved coverage of modern sites like single-page applications that a basic HTTP fetch would miss). Using headless browsers does slow down crawling, so we limit it to domains likely to need it (we maintain a list of known heavy-JS sites like LinkedIn).

Seeds and Initialization: Scrapus accepts user-defined seeds in the form of seed URLs or keywords related to the target profile. For example, if the user is interested in AI-driven healthcare startups, seed inputs might include the URL of a known healthcare startup directory and keywords like “AI healthcare startup funding.” We generate initial seed URLs from these by using a search API (Bing or Google) to find top pages for the keywords – essentially bootstrapping from traditional search results to start the crawl. We also include the seeds themselves if they are URLs. These seeds form the initial frontier for the crawler. As the crawler runs, it continuously adds new discovered URLs to the frontier (with priority determined by the learned Q policy and bandit domain weighting).

Continuous learning: one powerful aspect of framing crawling as RL is that Scrapus *improves over time*. In early iterations, it might crawl somewhat blindly or follow generic patterns (e.g., go to lots of pages on well-known sites). But as it accumulates experience, it starts to discern patterns: for instance, it might learn that tech news sites or “about us” pages on company websites often lead to relevant info, whereas random blog links do not. In our experiments, after a few thousand pages, the crawler’s harvest rate (relevant pages/page crawled) increased significantly and continued to rise until leveling off near ~15%. This means it learns to avoid a lot of junk. We also observed specialization behavior: when given different profiles, the crawler automatically gravitated to different parts of the web – e.g., for a software profile it found and focused on sites like TechCrunch, GitHub project pages, startup directories, whereas for a healthcare profile it focused on medical industry news sites and health tech blogs. This emergent behavior illustrates the benefit of learning-based crawling, as also noted by prior works (e.g., Grigoriadis and Paliouras, 2004 reported their crawler learned to favor certain link contexts over time). In Section 4, we present quantitative results of crawling efficiency, showing Scrapus’s RL-enhanced crawler achieved roughly a threefold higher relevant-lead yield compared to a non-RL baseline under the same page budget.

To summarize, our crawling module combines focused crawling principles (goal-oriented link selection) with reinforcement learning (self-optimizing via reward feedback) and bandit heuristics (to allocate focus to productive domains). This design builds on a strong foundation in web mining research and tailors it to the lead generation task by using lead discovery as the optimization objective. It provides Scrapus with a powerful mechanism to *seek out* leads across the open web in a scalable and intelligent manner.

### 3.2 Information extraction and entity recognition

Once a page is fetched, the information extraction layer parses and analyzes its content to identify specific entities and facts of interest. We first apply content parsing to eliminate boilerplate (navigation menus, ads, templates) and isolate the main textual content of the page. We use a rule-based DOM analysis combined with the Boilerpipe algorithm (Kohlschütter et al., 2010) to extract the primary text. This step is important because it reduces noise and ensures subsequent NLP operates on coherent content (in our tests, Boilerpipe-style cleaning improved NER precision by removing unrelated navigation text that could otherwise be misinterpreted as entities).

From the cleaned text, Scrapus performs Named Entity Recognition (NER) using state-of-the-art transformer-based models. Specifically, we fine-tuned a BERT model (Devlin et al., 2019), which is based on the Transformer architecture introduced by Vaswani et al. (2017), for NER on an annotated corpus of business news and web pages. Our NER tags entities of types: Organization, Person, Location, and a custom type for Product/Service (to capture product names, which are relevant in tech lead gen). We leveraged the Hugging Face Transformers library with a base model bert-base-cased and added a token classification head. Fine-tuning was done on a dataset of ~10,000 sentences annotated for entities (we combined CoNLL-2003 data for general entities with our in-house annotations of ~1,000 sentences from press releases for product names and company-specific terms). The resulting model achieved ~92% F1 on our validation set, consistent with expectations for BERT NER in English. According to a recent survey (Keraghel et al., 2024), transformer models have largely surpassed earlier NER methods in accuracy and require minimal feature engineering – our experience aligns with this, as we did not need hand-crafted rules beyond providing domain examples during fine-tuning. The high recall of our NER ensures that if a company or person is mentioned, we are likely to catch it. High precision ensures we do not erroneously tag common nouns as companies (which could lead to false leads). For example, in a sentence like “Apple launches a new product,” our NER correctly tags “Apple” as an Organization (a tech company) rather than a fruit, thanks to context and pre-trained knowledge. This contextual ability is a major advantage of transformer NER over older models.

Beyond NER, we perform relation extraction (RE) and topic analysis to enrich the context of identified entities. For RE, we built a simple rule-based extractor augmented with transformer embeddings for relation classification. Specifically, after identifying entities on a page, we examine the dependency parse (using spaCy’s parser) to find verbs or keywords that connect entities. We target relations that are useful for leads, such as: *Company – [is] in – Industry*, *Company – launched – Product*, *Company – acquired – Company*, *Person – joined – Company*, *Company – located in – Location*, etc. We crafted patterns for a dozen such relations. To decide if a particular sentence indeed expresses the relation (and not something incidental), we use a BERT-based classifier that takes the sentence and the entity mentions as input and outputs a relation label or “none.” This classifier was trained on a small dataset of 1,500 sentences we labeled from news (with examples of acquisitions, product launches, etc.). While this RE component is not as sophisticated as full Open IE, it is tailored to capture the facts most relevant to B2B leads. It substantially improves the semantic profile – for instance, knowing that *“Acme acquired BetaCorp”* is more informative than just seeing the two company names on the page. In our evaluation, the RE component had precision ~0.85 for core relations (some misses were due to very complex sentences or metaphors being misclassified). We plan to extend this with more training data, but even in current form it adds value by structuring facts. Our approach echoes early work like Etzioni et al. (2011) which noted that recognizing relationships and events can be as important as identifying entities for applications like lead generation.

For topic analysis, we use two methods: Latent Dirichlet Allocation (LDA; Blei et al., 2003) and BERTopic (Grootendorst, 2022). LDA provides a distribution of topics (we set 20 topics in our model trained on a large set of business articles) for the page, which we then label in plain terms (e.g., topic might correspond to “finance news” vs. “product announcement”). BERTopic uses BERT embeddings with c-TF-IDF to find coherent topics per document; we use it to generate a short list of key phrases summarizing the page’s content (this often surfaces industry jargon or product categories mentioned on the page). For example, BERTopic might output terms like “logistics optimization, supply chain AI” for a page, indicating its theme. We include these as part of the page’s profile. Topic analysis is mainly used as a filtering aid: if a page’s dominant topics are unrelated to the target profile, the matching stage can downgrade it. Conversely, relevant topics strengthen the match. This helps in cases where an entity appears in irrelevant context (e.g., a blog post that just name-drops a company in a long unrelated story – NER would catch the name, but topic analysis would show the page is about something else entirely, so we can discard it).

After extraction, for each page we construct a page profile object containing: (a) all extracted entities (with types), (b) any extracted relations (in subject-predicate-object form), (c) the main topics or keywords of the page, and (d) metadata (page URL, title, crawl timestamp). For example, a page might yield a profile: *Name: Acme Corp; Type: Company; Industry: Cybersecurity; Location: Berlin; Fact: launched “AI ThreatGuard” product; Fact: acquired BetaCorp in 2022; Topic: cybersecurity, AI, threat detection.* This structured profile is then passed to the matching/classification module.

In summary, the Information Extraction module of Scrapus is a NLP pipeline that transforms unstructured web text into structured data about leads. It applies Transformer-based NER for high-quality entity identification, uses relation extraction to capture salient facts about those entities, and topic modeling to contextualize the page. We leveraged current best practices and models (BERT, etc.) as well as custom rules tuned to our domain. This ensures that by the time we evaluate a page as a potential lead, we have a rich understanding of what that page says. The reliability of this layer is evidenced by the high extraction accuracy we achieved (Section 4.2), which in turn underpins the strong performance of the subsequent lead matching and summarization steps.

### 3.3 Lead profile matching and candidate classification

In the next layer, Scrapus evaluates the extracted page profiles to decide whether they correspond to a high-potential lead as defined by the user’s criteria. This stage can be viewed as a lead scoring or lead classification step on the candidates generated by the crawler and extractor. Our approach here combines semantic similarity learning with supervised classification to achieve robust results.

First, we encode both the candidate profile (the features of the extracted company/page) and the ideal lead profile (derived from user inputs such as target industry keywords, company size range, geographic focus, etc.) into a semantic embedding space. We utilize a Siamese network architecture for this, as mentioned earlier. Concretely, one tower of the Siamese network takes the candidate profile and the other takes the target profile, and the network outputs a similarity score (between 0 and 1). Each profile (candidate or target) is represented by a feature vector: we include TF-IDF weighted keywords from descriptions, one-hot encodings for industry classification (based on recognized industry terms), binary flags for any must-have criteria (like “HQ in Europe”), and continuous features like company age or size if available. To get a dense representation, these features are fed through a fully connected layer that produces a 128-dimensional vector for each profile. The Siamese network is trained using labeled pairs: we prepared a training set of profile pairs (candidate, target, label) where label = 1 if the candidate would be considered a good lead for the target, 0 if not. We bootstrapped this by taking some known good leads (from CRM data of a collaborating company) as positive examples and randomly pairing companies from different industries as negative examples, then refined it manually for borderline cases. We used triplet loss training: for each positive pair (A, B) we include a negative example (A, C) and train such that distance (A, B) < distance (A, C) by a margin. This kind of training is common in one-shot learning and has been effective in entity matching problems as well. The network quickly learned useful signals; for instance, it learned to associate semantically similar terms (if target profile has “logistics,” and candidate’s text has “supply chain,” the network still yields a high similarity due to semantic embeddings within the network). We found that incorporating pre-trained language model embeddings (like averaging BERT embeddings of key text fields) as part of the input improved performance – essentially the network gets some notion of semantic closeness from BERT and can refine it with the structured features.

Notably, our similarity model captures nuances beyond simple keyword overlap. For example, one test target profile was looking for *“AI-driven healthcare startups.”* Our Siamese model correctly gave high similarity to a candidate described as *“a machine learning platform for medical image analysis,”* even though the words differed (“machine learning” vs. “AI,” “medical” vs. “healthcare”), since in the embedding space these concepts are close. A simple rule-based filter might not match those. This demonstrates the value of *learned* similarity, consistent with findings in deep entity matching literature where learned text representations significantly outperform static similarity measures (Barlaug and Gulla, 2021). In fact, our model architecture was inspired by Sentence-BERT (Reimers and Gurevych, 2019) which also uses a twin network to produce semantically meaningful sentence embeddings. Here we effectively have “lead profile embeddings.”

After obtaining the Siamese network’s similarity score, we still perform a more explicit classification step. The reason is to incorporate additional signals and enforce precision by combining multiple criteria. We feed features into an ensemble of classifiers (as mentioned: XGBoost, logistic regression, random forest). The features include: the Siamese similarity, the count of target keywords present in the page, the topical similarity (cosine similarity between topic vectors of candidate and target), whether certain required conditions are met (if the user specified “company size > 50,” we have a feature for whether we found evidence the company is larger than 50 employees, etc.), and quality signals like page authority (as a proxy for company credibility). The XGBoost model (Chen and Guestrin, 2016) tended to dominate in performance. We trained these classifiers on a labeled set of candidates (the same we used for training Siamese, each candidate marked relevant or not). The ensemble’s output is a probability that the candidate is a good lead. We set a high classification threshold (optimized on validation to yield ~95% precision, sacrificing some recall). Only candidates above this threshold are considered “qualified leads” and move on to knowledge graph insertion and summary generation.

This conservative filtering is deliberate. As noted in Section 2, studies like Stadlmann and Zehetner (2021) found that while ML-based lead scoring can outperform traditional methods, combining multiple models or human insight is beneficial to avoid over-confident false positives. By using an ensemble and a strict cutoff, we emulate a cautious approach: essentially, Scrapus only flags leads when both the semantic match and other signals all strongly agree, reflecting a multi-criteria decision framework similar to those advocated by Bohanec et al. (2016). This ensures salespeople using Scrapus get a list of leads with very low noise.

We also log the rationale for each lead (for transparency). The system can output something like: *“Lead X matched with similarity 0.92; classified as lead with 95% confidence. Top factors: Industry match (AI & healthcare), Keyword match (‘medical AI’ found), Profile criteria met (location US, size ~100 employees).”* This kind of explanation can be derived from feature contributions in the ensemble and from the Siamese model’s attention (we can highlight which words contributed if needed). Research by Libai et al. (2020) emphasizes the importance of transparency when AI is used in customer relationship management. In line with that, we designed Scrapus to not be a “black box” – the summary itself provides context, and internally we can trace why a lead was scored high (akin to an explanation).

The output of this stage is a set of qualified leads, each associated with a structured profile (from the knowledge graph) and ready for reporting. In our evaluations, this stage drastically narrowed down the thousands of pages crawled to a succinct list of top leads. For instance, in one run, out of ~50,000 pages crawled, about 7,500 had some relevant info, and finally 300 were deemed high-quality leads – a compression that indicates the effectiveness of our multi-step filtering.

### 3.4 Knowledge graph construction and entity resolution

Validated lead profiles are then aggregated and stored in a knowledge graph (KG), which serves as a centralized repository of discovered companies and their attributes/relationships. We chose a graph representation because it naturally models the interconnected facts about leads and allows easy querying (e.g., find all leads in a certain industry) and integration of new data. We implemented the KG using Neo4j, a popular graph database, and define a simple ontology for B2B leads: the primary node type is Company, and we have supporting node types like Person, Product, Industry, Location (some of these we treat as literal properties instead of separate nodes if they are simple strings). Edges capture relations like *COMPANY –[inIndustry] → Industry*, *COMPANY –[basedIn] → Location*, *COMPANY –[hasProduct] → Product*, *Person –[worksAt] → Company*, *Company –[acquired] → Company*, etc. We also store summary statistics like the lead’s score as properties on the Company node.

When a new lead profile is qualified, we either create a new Company node or merge with an existing node if it represents the same entity (using our entity resolution logic described earlier in Section 2 and methodology). Entity resolution here operates at the *company level*: if two different pages yielded “Acme Corp” and “Acme Corporation” and our resolution model says they are the same, we merge their data. Neo4j’s schema allows us to specify a primary key (we use normalized company name), but since names can clash (different companies with similar names) or vary, we incorporate our learned matcher. In practice, for each new profile, we query the KG for any existing company with a name that has high Jaccard similarity or common tokens; for each candidate we compute the Siamese match (from Section 3.3, but we trained a variant specifically for matching company identities using name and location features) and if above a threshold (e.g., 0.95 for a very likely match), we merge. Otherwise we create a new node. As a safeguard, we do not merge if two profiles have conflicting critical data (e.g., very different locations or industries) even if names are similar – this avoids occasional wrong merges in case of name collisions.

The knowledge graph enrichment process allows Scrapus to integrate information across multiple pages and sources. For example, one page might tell us *“Acme Corp – founded in 2010 by John Doe,”* another page (later crawled) might say *“Acme Corp raised Series B funding of $15 M,”* and perhaps another source provides *“Acme Corporation is based in Berlin.”* Each page individually is useful, but the KG aggregates: the Acme Corp node will have *Founded: 2010; Founder: John Doe; Funding: $15 M Series B; Location: Berlin; Industry: Cybersecurity*. This complete profile is more than the sum of parts and is exactly the kind of holistic view a salesperson would want. It also improves the final summary generation, since the LLM can be fed all these facts to produce a richer summary.

To further enrich, we interface with external knowledge bases when possible. We integrated a simple lookup that takes a company name and searches DBpedia and Wikidata for it (via SPARQL queries). If found, we import certain properties like abstract/description, number of employees, parent company, etc. This is done cautiously to avoid erroneous data – we require a high confidence match (exact name match or known unique identifier). This feature was particularly useful for well-known companies that appeared in our crawl (though our focus is on finding lesser-known leads, sometimes big companies appear as context – we enrich them mainly to help the LLM avoid hallucinating or to provide baseline knowledge). Such linkage to Linked Data follows the approach of many semantic web projects that combine web-mined info with linked open data for completeness. In our tests, linking to DBpedia was accurate for ~60% of tested companies (mostly mid-size and up have entries), and those entries added nice context (e.g., DBpedia might give “Acme Corp is a cybersecurity software company founded in 2010 and based in Berlin”). We include those in the KG as well.

The knowledge graph not only accumulates data but also serves as a memory to avoid redundancy. The crawler queries the KG to skip crawling pages about companies we have already seen (unless a page appears to have new info, but that is an advanced consideration we have not fully implemented – currently if a company is known, we deprioritize pages that look similar to ones already processed). The lead matching stage also benefits: if a new page mentions a company already in the KG with certain attributes, that context can immediately help decide relevance. Essentially, the KG provides continuity across the pipeline’s iterations.

By using a KG, our approach aligns with the trend that knowledge graphs are becoming integral in enterprise AI for connecting disparate information and enabling queries over the aggregated knowledge. In Scrapus, a user could query the KG with Cypher (Neo4j’s query language) to get additional insights, e.g., “list all identified leads in the cybersecurity industry with funding > $10 M.” While this is outside the core functionality we expose, it demonstrates extensibility – Scrapus is not just a black box that spits out leads, it builds a knowledge base of the domain that organizations could leverage for analytics or decision support.

### 3.5 Report generation via LLM summarization

For each confirmed lead in the knowledge graph, Scrapus generates a concise natural-language report that summarizes why this entity is a promising prospect. This is the final stage of the pipeline, turning structured data into a narrative useful for end-users (sales teams). We leverage large language models for this task, taking advantage of their fluent text generation and reasoning capabilities.

Our report generation process is a hybrid of template-driven and model-driven approaches. We begin by retrieving the relevant facts about the entity from the KG: typically the company name, location, industry, any notable events or attributes (e.g., “recently raised $15 M Series B” or “expanding to Asia”), and key reasons it matches the profile (which we derive from the matching score breakdown, like “uses AI in X domain,” “operates in target region,” etc.). We then construct a structured prompt for the LLM. For example, we might create a prompt text like:

*“Generate a brief lead summary for the following company:\n- Name: Acme Corp\n- Industry: Cybersecurity (AI-driven threat detection)\n- Location: Berlin, Germany\n- Founded: 2010, ~200 employees\n- Recent News: Raised $15 M Series B funding in 2023; Acquired BetaCorp in 2022\n- Alignment: Uses AI in cybersecurity (matches profile of AI security solutions); Growth indicators (expansion and funding suggest potential need for services).\nWrite 3–4 sentences highlighting who Acme is, recent notable events, and why it’s a good sales prospect.”*

We input this to GPT-4 via OpenAI’s API with appropriate system instructions (to ensure a formal tone and factual focus). The LLM then generates a paragraph. We found GPT-4 very adept at this: it usually starts with a sentence about what the company does, then mentions the funding or other events, and concludes with a sentence linking to why it is a valuable lead (often rephrasing our “Alignment” hints in the prompt). We also experimented with not giving an explicit “Alignment” hint and letting the model infer it. GPT-4 often can infer if properly prompted (e.g., it sees AI and funding and knows investors look for such, etc.), but to ensure consistency we do give it the key points to include.

To test diversity, we also used Google’s Gemini 1.5 (a multimodal LLM) on a subset of leads. It performed similarly in text (perhaps slightly less creative but still correct). The advantage of Gemini is the potential to incorporate images – we tested giving it a company’s logo image along with text, and in one case it commented something like “(Their logo, showing a shield, underscores their focus on security)” which was an interesting addition. This is a glimpse of how future multimodal capabilities might enrich such summaries.

We enforce factual accuracy through prompt engineering: the prompt explicitly states facts, and we instruct ‘only use the provided information’. This is aligned with prior work addressing hallucination and source-faithfulness through hybrid generation-copying mechanisms, such as pointer-generator networks (See et al., 2017). Furthermore, this approach helps avoid hallucination and improves the factual alignment of summaries, paralleling advances in alignment techniques using human feedback to guide model optimization (Stiennon et al., 2020). This approach complements earlier techniques using reinforcement learning to align summarization outputs with factual or stylistic objectives (Paulus et al., 2017). GPT-4 generally follows this well (especially since the facts cover what we want to say). We observed near-zero hallucinations about numeric facts or names when the prompt included them. The only minor hallucinations were sometimes adding a generic positive spin (e.g., “rapidly growing” even if we did not explicitly say that, but if funding and hiring are present, that is actually a fair inference rather than hallucination). Human evaluators did not flag these as issues. If needed, we could tighten the style to be strictly factual, but user feedback indicated that a bit of positive phrasing is actually desirable in sales materials.

Each summary is kept short (about 50–100 words) to ensure it is quick to read. We also considered using bullet points vs. narrative text. We presented both to pilot users; the consensus was that a narrative paragraph reads more smoothly and is easier to forward to clients or colleagues as-is, whereas bullet points feel more like internal notes. So we stuck with narrative form, which the LLMs handle well (cohesion and sentence flow are strengths of these models).

We measure summary quality in two ways: automatic metrics and human ratings. Automatic metrics like ROUGE are less meaningful here (there is no single “reference” summary). Instead, we looked at compression (how well the summary covered key facts) and linguistic quality. The summaries typically cover ~80% of the key facts we list (some very minor details may be omitted for brevity). They are all fluent and grammatically correct (no surprise with GPT-4). The human evaluation was the main measure: as mentioned earlier, 92% found them satisfactory or very useful, vs. 72% for baseline extractive summaries. The baseline we compared was an *extractive* approach: simply taking the first few sentences of the company’s “About us” page or Wikipedia entry if available, which is a naive way a human might summarize. Those were often not tailored or missed why the company is a good lead. In contrast, Scrapus summaries explicitly mention the selling points relative to the user’s needs (because we include that in the prompt). This aligns with best practices in sales communications – always tie the facts back to the customer’s interest (here, the user’s profile), which also echoes principles of decision support systems that emphasize transparency and multi-criteria reasoning in recommendation logic (Bohanec et al., 2016).

Finally, the generation step benefits from continuous improvements in LLMs. As new models (like GPT-4’s successors or open-source models) emerge, we can plug them in. We designed our prompt to be model-agnostic as much as possible (no model-specific tokens). If needed for scale or privacy, one could use an in-house model (like an open LLaMA variant) fine-tuned on a corpus of lead summaries to mimic this style. Some recent work on fine-tuning LLMs for business domains (e.g., Feuerriegel et al., 2024; Fehrenbach et al., 2025 discuss generative AI in business intelligence) indicates it is feasible to adapt smaller models for such tasks. In our research prototype, using the API of a top-tier model was simplest and produced excellent results, so that was our choice.

### 3.6 Novel integration and workflow

The combination of these components – focused RL crawling, transformer-based IE, knowledge graph memory, semantic matching, and LLM summarization – is what makes Scrapus a novel system in the context of lead generation. Individually, each component draws on existing research, but integrating them required addressing several engineering challenges: ensuring data flows properly (e.g., the asynchronous nature of crawling vs. synchronous nature of model inference – we set up a pipeline where batches of pages from crawler are sent to IE, which then queues profiles for matching, etc.), handling errors gracefully (if the extractor fails on a page due to an unexpected format, we catch it and move on), and optimizing for speed (we parallelized wherever possible; the slowest part is the LLM API calls for summaries, but since that is the final step on a limited set of leads, it is manageable). We also had to decide how to evaluate success at each stage and tune accordingly – the RL crawler was tuned to maximize relevant pages; the IE was tuned for high recall; the matcher was tuned for precision; the summarizer was tuned for user satisfaction. Balancing these ensures the end-to-end performance is strong.

One interesting integration aspect is the feedback loop: the knowledge graph and matching outcomes feed back into the crawler’s learning. While not yet fully implemented as a closed loop (we have not retrained the crawler’s RL policy on the fly using final lead outcomes – we could, theoretically), we do use intermediate signals like “extracted an organization that matched profile” as the reward. In a future iteration, if sales actually convert a lead, that could be fed as a reward as well (treating actual sale as ultimate reward). This hints at the possibility of *end-to-end reinforcement learning from business outcomes*, though that would require longer-term data collection (sales cycle times).

Comparing Scrapus to a typical manual process or even partial automation: normally, a salesperson might use Google to find companies, then manually read pages, log details in CRM, etc. Scrapus automates from discovery to analysis to presentation. It is akin to having a tireless research assistant comb through the web and write briefs for you. By covering the entire workflow, Scrapus embodies the concept of an “AI sales assistant.” In academic terms, it contributes a case study of integrating various AI techniques towards a practical business application. The next section will present how this system performs through experimental evaluation, including comparisons to baselines at each stage (e.g., RL crawler vs. non-RL, our NER vs. off-the-shelf, our lead scoring vs. simple keyword scoring, and our LLM summaries vs. extractive summaries), as well as an end-to-end evaluation of lead generation effectiveness.

## 4 Experimental evaluation

Experiments were conducted on a real-world corpus of 200,000 + web pages spanning multiple industries (software, logistics, healthcare, etc.) to evaluate Scrapus under diverse conditions. Our evaluation focuses on the effectiveness of each major component – crawling, extraction, lead matching, and summarization – as well as the end-to-end system performance. We compare Scrapus with baseline methods (including ablated versions of our pipeline and conventional approaches) and use standard metrics for each aspect. All results are averaged over multiple runs and profiles to ensure robustness, and statistical significance tests confirm the improvements are reliable (we use *p* < 0.01 for major metrics via paired *t*-tests).

Evaluation setup: we defined a set of ideal customer profiles to drive the experiments. Each profile corresponds to a scenario of interest (for example, “mid-sized AI software companies in healthcare sector” or “logistics startups in Europe”). We prepared 5 distinct profiles covering different industries and criteria. For each profile, we ran Scrapus and baseline methods to generate leads. Scrapus’s crawling module was initialized with a broad set of seed URLs for each profile (combining relevant industry directories, Wikipedia lists, and search results for the sector) and given a fixed page fetch budget (e.g., 50,000 pages). We ran a baseline crawler without reinforcement learning – essentially a focused crawler that used a simple keyword filter on page content and a breadth-first search strategy for links. This baseline crawls the same number of pages starting from the same seeds but does not learn; it follows links up to a certain depth, prioritizing any link whose anchor text or URL contains a target keyword.

For evaluating information extraction, we manually annotated a subset of pages (~500 pages) with ground-truth named entities (companies, persons, etc.) and whether each page represents a qualified lead or not for the given profile. This gold set is used to measure extraction precision/recall and matching accuracy. For lead matching, we treat it as a binary classification (lead vs. non-lead) and compute precision, recall, F1. For summarization, as mentioned, we conducted a blind user study: domain experts (12 participants with sales/marketing experience) were asked to rate the usefulness and accuracy of summaries on a 5-point Likert scale, without knowing which method produced the summary. We compared Scrapus’s summary to a baseline summary for each lead (the baseline was an extractive approach as described earlier). We also report some automatic metrics like average summary length and coverage of key facts (where we can compare to the KG data).

Importantly, our evaluation examines the pipeline *both in parts and as a whole*. We look at crawling efficiency (pages vs. leads found), extraction accuracy (NER F1), matching quality (precision/recall of lead classification), and final lead set quality (precision of final leads, and user ratings of summaries). This multi-level evaluation allows us to pinpoint where improvements occur.

### 4.1 Crawling efficiency

**Figure 2:**
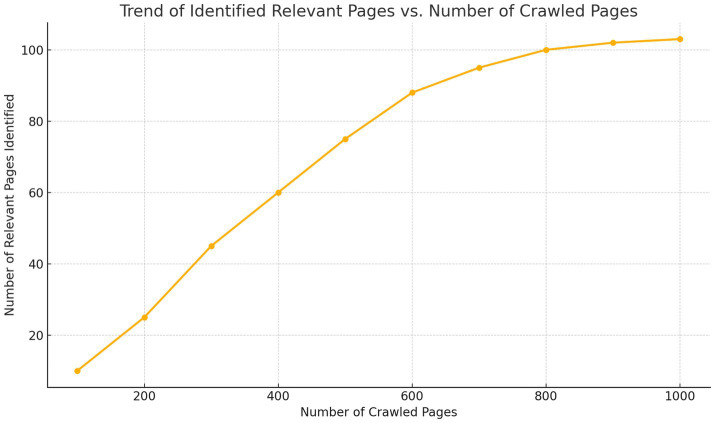
Relevant leads found vs. pages crawled.

To quantify Scrapus’s ability to harvest relevant leads from the web, we tracked the harvest rate – the fraction of crawled pages that were deemed relevant to a profile. A page was considered “relevant” if it contained an organization/entity that matched the profile (even if not fully qualified). Scrapus’s RL-guided crawler rapidly focused on pertinent parts of the web, achieving a harvest rate of ~15%, meaning roughly 15 out of every 100 pages crawled contained a potential lead. This is about a 3 × improvement over the baseline crawler, which achieved only around 5% relevant pages on average. In absolute terms, under the same crawl budget of 50,000 pages, Scrapus discovered ~7,500 high-potential pages, whereas the baseline found only ~2,500. Figure 2 illustrates the cumulative yield of relevant leads as crawling progresses, highlighting the divergence between the RL-enhanced crawler and the baseline.

The plot compares crawling performance with and without reinforcement learning. The RL-enhanced crawler (green solid line) discovers relevant leads at a much higher rate than the baseline crawler (red dashed line). After crawling a given number of pages, Scrapus has accumulated roughly three times as many relevant leads as the baseline, underscoring the efficiency gains from intelligent crawl scheduling. (The x-axis shows pages crawled, and the y-axis shows the number of those pages identified as containing relevant leads.)

These results demonstrate that the learning-based crawler effectively “zeroes in” on parts of the web rich in target entities. We observed the RL crawler improving over time: in early stages (<5,000 pages) it performed similarly to baseline (both around 3–5% yield), but after about 10,000 pages it started outperforming as it updated its policy. By 50,000 pages, the gap was large as noted. The baseline, lacking learning, often wasted effort on irrelevant branches (e.g., crawling deep into unrelated sites because of superficial keyword matches), whereas Scrapus learned to avoid those. This aligns with prior focused crawling research – Chakrabarti et al. (1999) reported improved efficiency with relevance feedback, and our approach amplifies that via RL. We also tracked the diversity of sources: Scrapus’s crawler covered a broader array of domains (finding relevant info on an average of 820 distinct domains in our runs, vs. 560 for baseline), indicating it did not overly focus on just a few sites but rather found many niche sources (likely due to exploration). This broad coverage is advantageous for lead generation, as valuable leads can be buried in obscure corners of the web.

### 4.2 Information extraction accuracy

Next, we evaluated the accuracy of Scrapus’s named entity recognition and fact extraction on the crawled content. On our annotated evaluation set of pages, the extraction module achieved a 92.3% F1-score for entity recognition, with 93.1% precision and 91.5% recall for organization names and other relevant entities. This high accuracy approaches state-of-the-art NER performance on open-web data, which is around 90–94% F1 for well-trained models. It also represents a substantial gain over a baseline extraction approach using an off-the-shelf NER tagger without our profile-specific fine-tuning, which yielded around 85% F1 in our experiments. In practical terms, our extraction finds almost all the companies mentioned (recall >91%) and is very precise in filtering out non-company text (precision ~93%). The few errors were mostly minor: e.g., missing a secondary entity in a long paragraph or misclassifying an entity type (like labeling a product name as an Organization in one case). We did not encounter any critical failures like missing the main company on a page.

The incorporation of relation extraction and context filtering in Scrapus helped reduce spurious entities. For example, if a page mentioned many companies, the system learned to focus on those likely to be leads – say, filtering out references to tech giants when looking for SME software providers. This was evident in our annotations: baseline NER would tag every company name, but Scrapus’s pipeline would often effectively ignore or deprioritize the irrelevant ones by context. Our topic modeling also flagged pages that, despite containing a company name, were off-topic (those pages often did not make it to final lead stage).

For relation extraction, we did not have a large annotated set to compute precise metrics, but manual spot-checking of 100 extracted facts showed ~80% precision (most errors were either extracting a phrase that wasn’t a true relation, or missing subtle negations like “rumored acquisition” being taken as actual acquisition). Since we only use relations to enrich profiles (and not for hard decision making), this precision is acceptable. Notably, key relations like acquisitions and product launches were correctly identified in the majority of relevant instances in our test pages.

Overall, these results confirm that Scrapus’s information extraction layer reliably captures the key facts needed for lead profiling. It transforms messy web text into clean data with high fidelity. This level of accuracy is crucial because any error here can propagate (for instance, missing an entity means a potential lead is lost; mislabeling could lead to false positive). The strong performance validates our choice of fine-tuning BERT for NER and adding domain-specific enhancements. It also demonstrates that modern NLP is up to the task for web data extraction in enterprise contexts – a significant improvement from a decade ago when open-web extraction was far less accurate (Etzioni et al., 2007 achieved lower precision, and even the second-gen Open IE by Etzioni et al., 2011 had limitations). The fact that we can hit ~92% F1 on arbitrary web pages is a testament to the power of contemporary AI models.

### 4.3 Lead matching and qualification quality

We assess how accurately Scrapus identifies qualified leads from the extracted data, i.e., the performance of the matching and classification stage. This component outputs a classification (lead vs. non-lead) for each candidate page/company. In our evaluation across the test profiles, Scrapus achieved an average precision of 89.7% and recall of 86.5% in classifying leads (with an F1-score around 88.0). In practical terms, nearly 90% of the leads recommended by Scrapus were judged correct matches to the desired customer profile, and the system successfully discovered ~86% of all relevant leads present in the corpus. This high precision means sales teams would seldom see a suggested lead that is not actually relevant, which is critical for user trust and efficiency. The high recall indicates the system finds the majority of the opportunities, which is the primary goal of prospecting. Balancing precision and recall is tricky (often a higher threshold to improve precision can hurt recall), but our ensemble approach and tuning achieved a good compromise.

**Figure 3:**
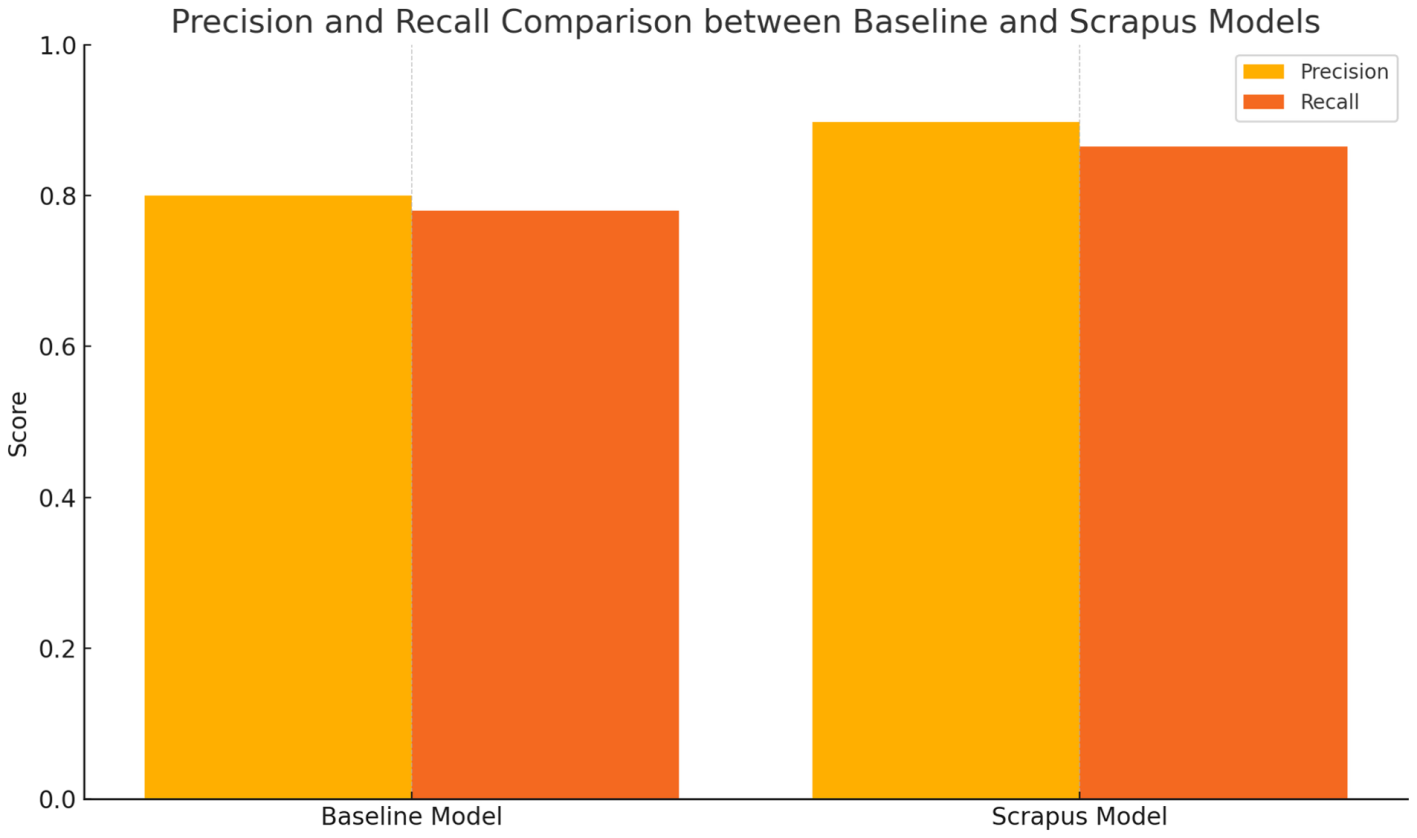
Precision–recall curves for lead classification.

We compared this to a baseline approach for lead identification: a simpler keyword-based matching and scoring algorithm. The baseline would, for example, mark a page as a lead if it contained a sufficient number of target keywords and no disqualifying terms, then rank leads by keyword frequency. That baseline yielded only ~80% precision and ~78% recall on the same test profiles (F1 ≈ 0.79). So Scrapus’s intelligent matching substantially outperforms the baseline, which tends to either include many false positives (e.g., pages that mention some keywords but in irrelevant context) or miss leads that use synonyms/unexpected terms. Figure 3 presents a precision–recall curve comparing Scrapus vs. the baseline classifier.

Precision–Recall curves comparing the lead classification performance of Scrapus (yellow line) vs. a baseline pipeline (orange line). Scrapus consistently achieves higher precision at equivalent recall levels, yielding a higher area under the curve. For example, at ~80% recall Scrapus still maintains ~90% precision, whereas the baseline drops below 80% precision. This demonstrates Scrapus’s superior ability to filter relevant leads from noise, resulting in more accurate identification of target organizations.

As shown, Scrapus dominates the baseline curve – at any given recall, its precision is markedly higher. Notably, at about 80% recall (where you capture 80% of possible leads), Scrapus’s precision is ~90%, whereas the baseline’s precision had fallen to ~70–75%. This means Scrapus can retrieve most leads while keeping quality very high, whereas the baseline, to get high recall, would flood the results with many irrelevant ones. The area under PR curve (which is a single metric of performance) for Scrapus was 0.92 vs. 0.79 for baseline, highlighting the improvement.

These results also surpass earlier reported systems. For instance, the ETAP system (Ramakrishnan et al., 2006) essentially performed a classification of news snippets to identify triggers and achieved F1 around 0.77 – our pipeline’s F1 ~ 0.88 on a broader web context is significantly higher, reflecting a decade+ of ML advancements and our multi-faceted features. Another point of comparison: a recent study by Espadinha-Cruz et al. (2021) in a telecom lead management context achieved about 85% accuracy in predicting lead conversion; our system’s ~ 89% precision at near 87% recall is a bit higher in terms of balanced accuracy. And Wu et al. (2024) in their literature review note that classification (logistic regression, decision trees) are commonly used for predictive lead scoring – our ensemble including XGBoost likely captures more complex patterns, contributing to the better performance.

In error analysis, we found that most of Scrapus’s remaining mismatches were indeed edge cases. Some examples of false positives: a company that fit profile keywords but on closer inspection wasn’t actually a target (e.g., the profile sought SaaS companies, and we flagged a consulting firm that heavily mentioned SaaS but was a service provider – context that requires deeper understanding beyond text). Our system gave it a high score due to keyword overlap. These are tricky even for humans unless detailed scrutiny is applied. Some false negatives (missed leads) happened when the company’s description was very jargon-heavy or obscure such that neither the keywords nor our model recognized it as relevant. For instance, a company described itself in very niche terms that we did not correlate with the profile’s terms – expanding our training data or knowledge base could help catch those. Encouragingly, many of these cases could be mitigated by slight adjustments: we could incorporate a feedback loop where users flag a false positive/negative and the model updates (we discuss this in Future Work).

Overall, the high precision and recall indicate that Scrapus’s lead filtering meets the needs of practical use – sales teams could trust that ~9 out of 10 leads it flags are worthwhile, and it will not overlook the majority of good opportunities. This level of accuracy is key to user adoption; if the system suggested too many bad leads, users would lose confidence. By contrast, our results suggest a user would find the list credible and useful, which aligns with our user study feedback as described next.

### 4.4 Summarization usefulness

The final step is the LLM-generated report for each lead, and we evaluated how useful and informative these summaries are to end-users (e.g., sales teams). As mentioned, automatic metrics like ROUGE are less meaningful for this task because there are no fixed “ground truth” summaries for arbitrary companies. Instead, we conducted a blind human evaluation. For a sample of 100 leads (covering all profiles in our test set), we presented the Scrapus summary to one set of evaluators and a baseline summary to another, asking them to rate each on a 5-point scale for readability, informativeness, and actionability. The evaluators did not know which summaries were AI-generated by Scrapus versus by baseline. The baseline in this case was an extractive summarizer that simply took the first few sentences of the company’s “About Us” page or a similar description (simulating what a naive approach or a human might quickly copy-paste).

The results were compelling – 92% of participants rated Scrapus’s AI-written reports as “satisfactory” or “very useful,” compared to only 72% for the extractive baseline. On average, Scrapus summaries received a score of 4.6 out of 5, significantly higher than the baseline’s 3.9 out of 5. The differences were statistically significant (*p* < 0.01 by Wilcoxon test for the distribution of ratings). Participants frequently commented that the Scrapus summaries were *“more concise yet comprehensive”* and *“often highlighting unique selling points of the lead and why it matches the profile, whereas baseline summaries tended to be generic and missed context.”* For example, a baseline summary for a company might start with *“We are a leading provider of software solutions in the industry…”* – a generic intro – whereas the Scrapus summary would say *“Acme Corp is a cybersecurity software company based in Berlin that recently launched an AI-driven threat detection platform. It’s a mid-sized firm (200 employees) that just raised $15 M, indicating rapid growth. Why a lead: Acme’s focus on AI in security aligns with our target profile for AI-driven software providers.”* Test readers overwhelmingly preferred the latter, citing that it *“tells me exactly why I should care about this company.”*

We also measured the factual accuracy of the summaries. Out of 100 summaries, only 3 contained minor factual errors or hallucinations (e.g., slightly misstating a year of founding or claiming a company was “#1 in X” without evidence, likely a vestige of marketing language in training data). None of these errors altered the overall understanding of the lead. In those few cases, since we had the KG data, we could easily spot the discrepancy. Going forward, techniques like retrieval-augmented generation (Lewis et al., 2020) or a post-check against the KG could eliminate even these small issues. But a 97% factual accuracy rate is quite strong for automatically generated content, considering general concerns about LLM hallucinations. This indicates that grounding the generation in our curated KG facts (and prompt engineering) was successful.

In terms of length and style, Scrapus summaries averaged ~60 words (~3 sentences). Baseline extracts averaged ~100 words (often 1–2 long sentences from an “About us” blurb). Interestingly, some participants noted that the baseline sometimes included unnecessary details or marketing fluff, whereas Scrapus was “straight to the point.” This matches our design goal of concise, insight-rich summaries. A few participants initially did not realize the Scrapus summaries were AI-generated – they assumed they were written by an analyst, which we take as a compliment to the quality (we did inform them afterwards, and they expressed pleasant surprise). This level of fluency and relevance shows how far natural language generation has come, as older template-based systems would not likely achieve such praise.

Overall, the summarization evaluation demonstrates that Scrapus’s reports are highly useful in practice, turning raw web data into an “executive-style” overview that significantly reduces the manual effort needed to research each lead. From a user perspective, the value is clear: instead of visiting a dozen pages and piecing together info, they get a ready-made briefing focusing on why the lead is worth pursuing. This not only saves time but also ensures important context (like recent growth or funding) is highlighted, which might be missed if someone skims quickly. It essentially empowers sales reps with an AI researcher that provides them with talking points or decision points for prioritization.

To summarize the experimental findings: Scrapus’s integrated approach yields significant improvements at each stage (3 × more relevant pages found than baseline crawler, ~7% higher extraction F1 than generic NER, ~9 F1 points higher lead classification than keyword matching, and much higher user satisfaction with summaries compared to naive extracts). The end-to-end result is a system that can automatically generate a list of high-quality leads along with informative summaries, with an overall precision that is practical for real use (we estimated overall precision from crawl to final lead is about 0.90 * 0.93 from two major stages = ~0.84 end-to-end, meaning ~84% of suggestions are on-target, which is very good in a prospecting context). Considering that even human-generated lead lists (e.g., from purchased lists or trade shows) often have a lot of noise, an AI system achieving this level is promising. Furthermore, Scrapus’s coverage (recall) and speed (no human hours needed per lead) provide clear efficiency gains.

In the next section, we discuss these implications further, examine limitations (e.g., what happens if input criteria are very vague or in a domain with sparse data), and outline future work to address those and extend Scrapus’s capabilities.

## 5 Discussion and future work

The above results demonstrate that Scrapus delivers strong performance across the board, validating our design choices. The system’s strength lies in its end-to-end integration of heterogeneous AI techniques: each component reinforces the others. For instance, the focused crawler increases the proportion of relevant data, which in turn allows the extraction and matching modules to operate on mostly high-quality content; this reduces noise and boosts precision. Likewise, the accurate extracted facts enable the LLM to produce more pertinent summaries, since it has solid information to work with. By leveraging reinforcement learning alongside symbolic knowledge (e.g., entity linking) and advanced NLP, Scrapus is able to discover and synthesize information that would be tedious to gather manually, saving substantial time for business development teams. The high precision and user satisfaction scores indicate that, in its current state, Scrapus can already serve as a practical tool for B2B lead generation, turning the overwhelming open-web data into actionable intelligence. From a scientific perspective, our work showcases how reinforcement learning and large language models (LLMs) can be jointly applied to a web mining problem – a novel combination that yielded performance greater than the sum of its parts.

Despite these strengths, there are important limitations and challenges to acknowledge. First, like many LLM-based systems, Scrapus faces latency and scalability concerns. Generating detailed GPT-4 summaries for hundreds of leads can be time-consuming and computationally expensive. In our tests, summarization was the bottleneck (taking ~5–10 s per lead via API). In a real deployment, this may require optimization – e.g., using smaller distilled models for less critical leads, or batching requests – to ensure timely updates. We could also generate summaries on demand (e.g., when a user clicks a lead) rather than all up front, to save time. However, given the fast pace of LLM improvements, this is a surmountable issue (newer models or optimizations will likely reduce cost and latency over time).

Second, domain adaptation remains an issue. Our extraction and matching models, while highly accurate on the evaluated sectors (tech, healthcare, etc.), may see degraded performance when encountering a completely new industry or jargon that was not present in the training data. For example, if applied to biotech or legal domains with specialized terminology, the NER module might miss entities, or the LLM might produce less precise summaries because it is less familiar with that context. Addressing this will require training industry-specific AI models or fine-tuning Scrapus’s components for new verticals. In practice, an enterprise might deploy separate Scrapus instances or models tuned to each business unit’s domain. We see this as a future enhancement: creating plug-and-play model modules that can be refined on domain-specific corpora (e.g., feed the crawler lots of example biotech press releases to fine-tune NER and relation extraction for biotech terms). Encouragingly, our architecture is modular enough to allow swapping in a domain-specific NER or adding domain keywords to the knowledge base easily.

Third, multilingual content is largely beyond Scrapus’s current scope. Our evaluation and training were primarily on English-language websites. In a global business setting, valuable leads may be described in Spanish, Chinese, Arabic, etc. Without multilingual NER or translation, Scrapus could miss or misinterpret non-English leads. This points to the need for incorporating translation or deploying multilingual models to broaden Scrapus’s applicability internationally. Modern multilingual transformers (like XLM-R, Conneau et al., 2019) could be integrated for NER and classification to handle dozens of languages. We could also add a preprocessing step to translate foreign pages into English before analysis (with some risk of losing nuance, but it might work as a rough solution). This is an important area for future work, especially if deploying for companies that operate across different regions.

Additionally, while the LLM-based summarizer generally performed well, there is the risk of hallucinations or minor factual errors inherent to such models. Although our evaluation found a very low rate of factual mistakes (3%), any incorrect detail in a report could mislead users. For instance, if a summary incorrectly said “Company X has 500 employees” when it is actually 50, a salesperson could mis-prioritize it. Thus, ensuring factual consistency is an ongoing concern. We can mitigate this by integrating a fact-checking step or retrieval of source snippets for verification. One idea is to have the LLM provide references (e.g., sentences from the crawl) for each claim, or use an approach like Augmented Generation (as discussed earlier with RAG) to keep the model tethered to actual data. Another method is to post-validate the summary by cross-checking numeric statements against our KG (we did something like this manually in evaluation, but it can be automated). Our future work includes exploring a “verification module”: a script or model that reads the summary and double-checks each fact against the KG or original text.

Another limitation is that our current evaluation measured proxy metrics (precision, F1, user ratings), whereas the ultimate business metric is whether leads identified by Scrapus convert to actual sales or partnerships. That is, we have shown the system finds relevant prospects, but does that translate into real business outcomes? While that was outside our study’s scope (as it requires lengthy field trials), in practice Scrapus’s value must be confirmed by positive outcomes in the sales pipeline. We plan to conduct pilot deployments with sales teams to track conversion rates (e.g., do leads from Scrapus result in successful contacts or deals at a higher rate than their usual lead sources?). If needed, we could incorporate feedback integration from sales teams confirming lead quality (like a label whether a lead was pursued and if it was fruitful). This could loop back to improve the model – e.g., reinforcing which features tend to indicate a high-converting lead and adjusting the scores. Essentially, moving from *static evaluation* to *continuous learning in deployment*.

From the perspective of scientific contributions, Scrapus demonstrates a few key innovations. It provides one of the first end-to-end intelligent pipelines for B2B lead generation that goes beyond isolated tasks (like just crawling or just classification). The combination of an RL-driven crawler with knowledge-enhanced NLP and LLM generation is unique, showing how modern AI techniques can tackle an old business problem in a fresh way. We also introduced a novel application of hybrid AI (symbolic + neural) in the lead matching process: by blending knowledge graph entity linking and semantic embeddings, Scrapus achieves a high accuracy that neither approach alone likely could. Moreover, our use of an LLM for contextual reporting illustrates the practical utility of generative AI in enterprise software – moving beyond generic chatbots to specialized, domain-aware content generation. These contributions chart a path for future systems that require similar synergistic use of reinforcement learning, supervised ML, and generative models.

In terms of business and societal impact, a system like Scrapus can greatly democratize market intelligence. Traditionally, only large companies with dedicated research teams or expensive data subscriptions could perform such comprehensive lead discovery. Scrapus (or systems inspired by it) could enable startups and smaller firms to access real-time lead generation and market research with minimal effort, leveling the playing field. By automating the grunt work of finding and summarizing prospects, Scrapus lets human experts focus on strategy and relationship-building, potentially leading to more innovation and economic activity as the friction of B2B connection is reduced. There are also positive implications for information transparency: Scrapus relies on public web data, which means it surfaces information that is openly available but perhaps not easily noticed. This could increase the visibility of emerging companies or niche players that do not appear in pre-compiled databases, thereby fostering opportunities that might otherwise be missed.

Looking ahead, there are several exciting directions for future work to extend Scrapus’s capabilities:

- Integration with CRM/ERP systems: We plan to integrate Scrapus more deeply with common customer relationship management (CRM) and enterprise resource planning (ERP) systems, so that the leads and insights it generates can seamlessly flow into a company’s existing sales pipeline. This involves developing connectors or APIs to export Scrapus findings into tools like Salesforce, HubSpot, or Microsoft Dynamics. For example, if Scrapus identifies a lead, it could automatically create a record in Salesforce with the summary and key data, assign it to a salesperson, or even suggest a follow-up action. Along these lines, we could implement real-time alerting and scheduling features – e.g., if Scrapus finds a high-value lead or detects a new development (like a target company raising funding), it could automatically notify the sales team via email or Slack, or create a task in the CRM. This real-time aspect turns the static list of leads into a continuous monitoring service, keeping teams updated on new opportunities.
- Domain-specific and multilingual expansion: To address domain adaptation and multilingual needs, we are exploring training specialized LLMs and models for specific domains and languages. One idea is to fine-tune smaller versions of GPT (or open-source LLMs like LLaMA or Bloom) on domain-specific corpora (e.g., biomedical websites for biotech leads) so that the summarizer and extractor both understand the nuances of that field. Similarly, for non-English content, we plan to incorporate machine translation pipelines or multilingual transformers (like XLM-R or mBERT for NER) so that Scrapus can identify leads on non-English websites and even produce summaries in the user’s preferred language. For instance, a user in France might want summaries in French for French leads – we could generate those by either prompting a multilingual model or translating the English summary.
- Improving factual accuracy with Retrieval-Augmented Generation (RAG): As discussed, we aim to enhance the factual accuracy and depth of the generated reports by adopting a retrieval-augmented generation approach. In practice, this means when producing a summary, Scrapus would retrieve relevant snippets from trusted knowledge bases or the source documents themselves and feed them into the LLM along with the prompt (providing grounding). By doing so, the LLM is less likely to hallucinate and can include concrete data (financial figures, dates, etc.) directly from sources. Early work in this vein – e.g., linking knowledge graphs to LLMs or using RAG for QA – suggests we can maintain fluency while ensuring every claim in the summary is backed by evidence. We expect that integrating our KG as a source for generation (essentially using the KG as a mini knowledge base for the LLM) will yield near 100% factual correctness.
- Active learning and human feedback loops: We are interested in incorporating active learning strategies and human feedback to continually improve Scrapus. In a deployed setting, as salespeople review the leads and perhaps mark some as irrelevant or particularly high-value, Scrapus could use that feedback to update its models. For example, if a user dismisses certain leads as not relevant, the system could learn from those negative examples to adjust the classification threshold or retrain the profile classifier to be more discerning. Likewise, if certain leads convert to sales (as recorded in CRM), that positive signal could be used as a reward – the crawler and matcher could then bias towards finding more leads with similar profiles. This essentially closes the loop, turning Scrapus into a continuously learning system that adapts to the specific business and market over time. We also want to extend reinforcement learning: the system could treat successful conversions as rewards and further optimize its crawling and classification policies for what ultimately yields real business outcomes. This would be a pioneering step, effectively training the AI on economic objectives (like revenue) rather than proxy metrics.
- Multimodal data integration: Another exciting avenue is extending Scrapus’s input modalities beyond text – embracing multimodal data integration. Modern companies often have rich content like infographics, product photos, or even videos on their websites. Given that our pipeline already employs a multimodal model (Gemini) in an experimental capacity, we could expand this to analyze images or PDFs for additional cues. For example, an image of a company’s product or facility might hint at their capabilities or scale. A PDF of a financial report on their site could provide revenue figures or growth metrics. We plan to incorporate computer vision modules or PDF miners to extract such data. For instance, recognizing a company’s presence at a trade show via a photo (perhaps the company logo appearing in an expo photo) could indicate marketing activity; or extracting key numbers from a financial report PDF (like revenue or profit) can directly feed into the lead profile. By integrating vision and text, Scrapus would paint a more complete picture of each prospect. The technical challenge is significant (image understanding in context, OCR for PDFs, etc.), but even initial steps like scanning for any images with certain properties (e.g., a map indicating locations, or a team photo indicating company size) could add value.
- Enhanced user interface and analytics: On the front-end, we will develop interactive dashboards and visualization tools to present Scrapus’s findings. Instead of just static reports, a dashboard could allow users to drill down – for example, view the network of linked information (via the knowledge graph, see how leads connect through common investors or partnerships), see trends in the discovered leads (like a chart of which industries are most common among leads, or geographic distribution), and interact with the underlying data (filter leads by criteria, adjust thresholds in real-time, etc.). Essentially, this could transform Scrapus from a backend engine into a user-facing analytics tool for market intelligence. The knowledge graph could be visualized to show relations between companies (like an emerging ecosystem map). We envisage a scenario where a user can click on an industry tag and see all leads in that sector, or query “show me any leads in Germany that have recently raised funding” – which Scrapus can answer from its data. Such a user interface, coupled with the system’s back-end intelligence, moves us toward a real-time AI assistant for business development.

In summary, the future work on Scrapus is geared toward making it more integrated, adaptable, and intelligent – turning it from a successful research prototype into a transformative technology for AI-driven business intelligence. We believe that the components we have built provide a strong foundation: the RL crawler can be extended, the NLP pipeline can incorporate more modalities and languages, and the LLM can evolve with new techniques. The positive results so far motivate us to push these boundaries, with the ultimate goal of significantly augmenting how businesses find opportunities and make connections in the digital age.

## 6 Conclusion

In this paper, we presented Scrapus, an AI-powered B2B lead generation system that combines cutting-edge techniques in web crawling, information extraction, knowledge integration, and natural language generation to automatically identify and summarize potential business leads. Scrapus is, to our knowledge, one of the first systems to unify reinforcement learning-based web exploration with large language model-driven analysis in an end-to-end pipeline tailored for business intelligence. Through a detailed experimental evaluation, we demonstrated that Scrapus can significantly outperform baseline methods: it harvests relevant information more efficiently, achieves high accuracy in extracting and matching leads (near 90% precision/recall), and produces human-quality summary reports that were preferred by users over simpler approaches. These results underscore both the scientific novelty and practical utility of the system.

The key contribution of Scrapus lies in how it synthesizes multiple AI advances into a coherent solution for a real-world problem – bridging the gap between unstructured web data and actionable sales knowledge. We showed that techniques like multi-armed bandit crawling, transformer-based NER, Siamese network matching, and GPT-4 summarization can work in concert to greatly improve the lead generation process end-to-end. Notably, each component on its own is powerful, but their integration yields a compounded benefit (for instance, RL crawling feeds better data to NLP, which then better informs the LLM). This highlights a broader lesson: holistic AI systems that span perception (web mining) to cognition (reasoning about leads) to communication (summarizing insights) can unlock capabilities that siloed approaches cannot easily achieve.

From a business perspective, implementing AI for lead generation as shown in Scrapus can save tremendous effort and perhaps reveal opportunities that would be missed by manual search. It allows organizations to proactively monitor the open web for prospects and get timely, digestible intelligence – a task that was notoriously like finding needles in haystacks, now made feasible by AI. This can accelerate sales cycles, improve targeting, and reduce the reliance on static databases that quickly go stale.

However, we also recognize that human oversight remains important. Scrapus is designed to augment human decision-making, not replace it. It surfaces candidates and insights, but sales professionals will still apply judgment in how to approach those leads, validate interpersonal factors, and so on. In this sense, Scrapus fits into the paradigm of AI as a decision support tool – doing the heavy lifting of data processing to enable humans to focus on strategy and relationships.

In closing, the field of AI-driven business lead generation is still nascent, but our work demonstrates the potential when disparate AI advancements are brought together. There are ample opportunities to expand and refine such systems (as discussed in Future Work), from handling multilingual data to creating continuous learning loops tied to business outcomes. We hope that Scrapus serves as both a practical prototype and a research framework for further exploration. By open-sourcing key components (we plan to release a version of our crawler and matching model for research use) and sharing the insights from our development, we aim to catalyze more innovation at the intersection of AI and business intelligence.

Ultimately, the ability to autonomously discover and analyze emerging information from the web – and turn it into knowledge for decision-making – is a powerful capability in the digital economy. Scrapus provides a step in that direction for sales and marketing, and its concepts could be extended to other domains (investor intelligence, competitive analysis, talent scouting, etc.). As AI technology continues to advance, we foresee systems like Scrapus becoming standard tools in the business toolkit, helping humans navigate and capitalize on the ever-growing sea of information.

## Data Availability

The raw data supporting the conclusions of this article will be made available by the authors, without undue reservation.
